# Social-Ecological Predictors of Opioid Use Among Adolescents With Histories of Substance Use Disorders

**DOI:** 10.3389/fpsyg.2021.686414

**Published:** 2021-07-16

**Authors:** Lindsey M. Nichols, Jonathan A. Pedroza, Christopher M. Fleming, Kaitlin M. O’Brien, Emily E. Tanner-Smith

**Affiliations:** ^1^Department of Counseling Psychology and Human Services, University of Oregon, Eugene, OR, United States; ^2^Prevention Science Institute, University of Oregon, Eugene, OR, United States

**Keywords:** opioid misuse, adolescence, substance use recovery, social-ecological model, SUD treatment

## Abstract

Adolescent opioid misuse is a public health crisis, particularly among clinical populations of youth with substance misuse histories. Given the negative and often lethal consequences associated with opioid misuse among adolescents, it is essential to identify the risk and protective factors underlying early opioid misuse to inform targeted prevention efforts. Understanding the role of parental risk and protective factors is particularly paramount during the developmental stage of adolescence. Using a social-ecological framework, this study explored the associations between individual, peer, family, community, and school-level risk and protective factors and opioid use among adolescents with histories of substance use disorders (SUDs). Further, we explored the potential moderating role of poor parental monitoring in the associations between the aforementioned risk and protective factors and adolescent opioid use. Participants included 294 adolescents (*M*_*age*_ = 16 years; 45% female) who were recently discharged from substance use treatment, and their parents (*n* = 323). Results indicated that lifetime opioid use was significantly more likely among adolescents endorsing antisocial traits and those whose parents reported histories of substance abuse. Additionally, adolescents reporting more perceived availability of substances were significantly more likely to report lifetime opioid use compared to those reporting lower perceived availability of substances. Results did not indicate any significant moderation effects of parental monitoring on any associations between risk factors and lifetime opioid use. Findings generally did not support social-ecological indicators of opioid use in this high-risk population of adolescents, signaling that the social-ecological variables tested may not be salient risk factors among adolescents with SUD histories. We discuss these findings in terms of continuing care options for adolescents with SUD histories that target adolescents’ antisocial traits, perceived availability of substances, and parent histories of substance abuse, including practical implications for working with families of adolescents with SUD histories.

## Introduction

Opioid misuse, broadly defined as the intentional use of opioids not directed by a prescriber, is a major public health concern in the United States, particularly among adolescents. In 2018, an estimated 699,000 (2.8%) of U.S. adolescents aged 12–17 reported past year opioid misuse and 169,000 reported past month misuse (Substance Abuse and Mental Health Services Administration, 2019). In 2019, the Centers for Disease Control and Prevention’s 2017 Youth Risk Behavior Surveillance Survey–a nationally representative survey that provides data of 9th through 12th grade students in public and private schools in the United States–found that approximately 14% of U.S. adolescents reported ever misusing opioids (Bhatia et al., 2020). Although U.S. adolescents aged 12–17 are less likely to report opioid use compared to older age groups (Back et al., 2010), adolescence represents a critical developmental stage for initiation of drug use, characterized by increased risk-taking as well as novelty and sensation seeking behaviors. Adolescents are at increased susceptibility to drug use and drug-related risks due in part to the salient influence of peers in conjunction with critical cortical development that occurs during this developmental period (Crews et al., 2007; Dayan et al., 2010; Romer, 2010; Winters and Arria, 2011). Further, early initiation of substance use and related risk behavior patterns increases risk for more progressive forms of substance use into adulthood (Chassin et al., 1999; DuRant et al., 1999; Lynne-Landsman et al., 2010; Van Ryzin et al., 2012). Thus, understanding salient risk factors associated with opioid use during this critical developmental period is paramount.

Adolescent opioid misuse has been associated with increased risk for negative outcomes into adulthood, including subsequent substance use disorders (SUDs) and more severe forms of drug misuse, including use of more potent opioids, such as heroin (Muhuri et al., 2013; Cerdá et al., 2015; Miech et al., 2015; Palamar et al., 2016b; McCabe et al., 2019). Compared to adolescents with cannabis or alcohol use disorders, those with opioid use disorders may also exhibit poorer long-term prognoses, including higher rates of school drop-out and multiple SUDs (Subramaniam et al., 2009; Godley et al., 2017). Among U.S. high school students, non-medical prescription opioid use is associated with increased odds of engaging in concurrent risky behaviors, including risky driving behaviors, violent behaviors, risky sexual behaviors, substance use, and suicide attempts (Bhatia et al., 2020). Given the wide-ranging short- and long-term consequences of adolescent opioid use, it is essential to identify the malleable risk and protective factors underlying early opioid misuse to develop more effective preventive interventions.

Adolescents with longstanding histories of excessive substance use or SUDs are considered a high-risk subpopulation who are particularly vulnerable to developing opioid use disorders and experiencing subsequent consequences. For instance, adolescents with histories of SUDs report high rates of comorbid mental health problems (Tanner-Smith et al., 2019) and high risk of relapse following SUD treatment (Cornelius et al., 2003; Chung and Maisto, 2006). Few existing studies have explored opioid-specific outcomes in this high-risk subpopulation, but there is some evidence that youth with SUDs who have received SUD treatment in the United States report high rates of opioid misuse (e.g., Osgood et al., 2012). Opioid misuse has been shown to be prevalent among adolescents in substance use treatment and was associated with an increased likelihood of having three or more co-occurring SUDs (Al-Tayyib et al., 2018). And among students who attended a recovery high school (RHS)–a form of continuing care for youth discharged from SUD treatment–78% reported ever using opioids/narcotics, compared to 13% in a national sample of students who received SUD treatment in the United States who were not enrolled in an RHS (Tanner-Smith et al., 2018). Further, prior research on youth with SUDs attending RHSs reported that among those who use heroin, 80% identified prescription opioid misuse as a precursor to heroin use (Vosburg et al., 2016). These findings demonstrate the unique risk profiles of adolescents with SUD histories and underscore the importance of identifying social-ecological risk and protective factors for opioid misuse specifically for this vulnerable subpopulation.

### Social-Ecological Predictors of Adolescent Opioid Misuse

The social-ecological model (Bronfenbrenner, 1979, 1994) is a comprehensive conceptual framework for understanding human development and is uniquely suited for examining risk and protective factors for adolescent opioid misuse (Twombly and Holtz, 2008; Jalali et al., 2020). The social-ecological model posits that human development and behavior are shaped by bidirectional relationships and interactions between an individual and five different environmental systems (microsystem, mesosystem, exosystem, macrosystem, and chronosystem). Particularly salient to identifying actionable mechanisms of adolescent substance use are those more proximal ecological systems, including individual characteristics (e.g., mental health, substance use history); microsystemic (e.g., peer/family substance use history, influence of family/peers); and exosystemic relationships (e.g., access and availability to illicit substances, school). Given the influence and importance of social contexts in adolescents’ lives (e.g., school, parents, peers), as well as bidirectional influences of these factors, the current study uses this guiding framework to examine a range of social-ecological predictors of adolescent opioid use and their interactions with parenting behaviors.

Extending from the social-ecological model, prior empirical research has found strong evidence for diverse ecological factors predictive of substance use and other related behaviors in adolescence across diverse populations (Arthur et al., 2002; Bränström et al., 2008; Cleveland et al., 2008; Hemphill et al., 2011). Among individual-level predictors, prior tobacco, marijuana, and alcohol use have been consistently identified as salient indicators for subsequent opioid misuse among the general adolescent population (Sung et al., 2005; Back et al., 2010; Palamar et al., 2015; Vaughn et al., 2016; Barnett et al., 2019; Griesler et al., 2019; Bhatia et al., 2020; Bonar et al., 2020; Osborne et al., 2020). Specifically, the odds of reporting having ever misused opioids were three times higher among adolescents with histories of alcohol use (vs. those without), and two times higher among those with histories of cigarette and marijuana use (vs. those without; Barnett et al., 2019). Additionally, specific mental health concerns, such as depression and anxiety (Schepis and Krishnan-Sarin, 2008; Young A.M. et al., 2012; Edlund et al., 2015; Monnat and Rigg, 2016; Chan and Marsack-Topolewski, 2019; Griesler et al., 2019; Bonar et al., 2020); post-traumatic stress (McCauley et al., 2010; Mackesy-Amiti et al., 2015); and antisocial behavior (Sung et al., 2005; McCauley et al., 2010; McCabe et al., 2012; Young A.M. et al., 2012; Edlund et al., 2015; Nargiso et al., 2015; Vaughn et al., 2016; Bonar et al., 2020) were associated with increased likelihood of adolescent self-reports of opioid misuse.

Within the microsystem, peers and parents are critical agents of socialization and influence in adolescents’ lives. The peer context contains some of the most robust predictors of adolescent substance use (Bauman and Ennett, 1994). Specifically, peer attitudes favorable toward substances are a consistent predictor of opioid misuse in the general adolescent population (Ford, 2008; Conn and Marks, 2014, 2017; Ford and Rigg, 2015; Nargiso et al., 2015; Vaughn et al., 2016; Schaefer and Petkovsek, 2017). In a nationally representative sample of youth ages 12–17, adolescents who associated with peers that use drugs or had attitudes favorable of drug use were approximately 1.4 times more likely to endorse non-medical prescription drug use compared to peers without these peer associations (Ford, 2008). Although the influence of peers on substance use increases during adolescence, the role of parenting continues to serve as a salient factor in predicting adolescent substance use involvement. Parenting factors, including poor parental monitoring, lack of parental involvement, parental histories of substance use, and tolerant parental attitudes toward substance use are associated with adolescent substance use, including opioid misuse (Sung et al., 2005; Gilson and Kreis, 2009; Donaldson et al., 2015; Edlund et al., 2015; Nargiso et al., 2015; Vaughn et al., 2016; Griesler et al., 2019; Bonar et al., 2020). Although peers and parents serve as important risk and protective factors, prior research has documented complex interactions between peer associations and parental monitoring, such that the substance use risk associated with peers may be magnified when adolescents experience low levels of parental monitoring (Kiesner et al., 2010).

Prior research has also identified several influential school and community-level (i.e., exosystem) risk factors for adolescent opioid misuse. Relevant school-level risk factors include academic achievement (Young A.M. et al., 2012; Veliz et al., 2013; Nargiso et al., 2015; Vaughn et al., 2016; Schepis et al., 2018; Barnett et al., 2019; Bonar et al., 2020) as well as school bonding and negative attitudes toward school (Ford, 2009; Young A.M. et al., 2012; Ford and Rigg, 2015; Nargiso et al., 2015; Nicholson et al., 2016). In a systematic review of studies on youth non-medical prescription drug use, five of six studies assessing low academic performance, school dropout, or lack of school-bonding found a significantly higher prevalence of prescription drug use among youth with these risks (Young A.M. et al., 2012). Relevant community level risk factors for adolescent opioid misuse include (perceived) availability and access to drugs in the community (Nargiso et al., 2015; Monnat and Rigg, 2016). In a nationally representative study of adolescents, perceived ease of access to illicit drugs was associated with 1.03 times greater odds of prescription opioid misuse (Monnat and Rigg, 2016). This body of literature thus demonstrates how diverse social-ecological systems can contribute to adolescent opioid use outcomes.

Parental monitoring is perhaps the most widely studied family risk factor for adolescent substance use. Prior research has found that low levels of parental monitoring moderate the associations between some community level risk factors (e.g., exposure to violence; Burlew et al., 2009; Udell et al., 2017), peer risk factors (e.g., substance using with peers; Kiesner et al., 2010), and individual characteristics including impulsivity (Haas et al., 2018) and depression (Geisner et al., 2018). Low levels of parental monitoring may thus exacerbate the relation between relevant social-ecological risk factors and substance use among adolescents. However, no research to date has examined parental monitoring as a moderator of the relationship between ecological risk and protective factors and opioid misuse among adolescents with SUD histories.

Despite the extensive body of evidence on risk and protective factors for adolescent opioid misuse, to date there has been limited evidence examining these associations in clinical samples of adolescents who may be at particularly high risk for opioid misuse (Bonar et al., 2020). Most prior research on this topic has analyzed data from large national surveys of U.S. adolescents, which can yield valuable insights on patterns in the general adolescent population; however, these findings may not be generalizable to high-risk adolescent subpopulations, such as those with SUDs. Among adolescents with SUD histories, the family environment, parental support, and involvement may be uniquely important for sustaining recovery and abstinence (Godley et al., 2005; White et al., 2009; Sussman, 2011; Fisher, 2014; Winters et al., 2018; Botzet et al., 2019). Given the important role of parents in adolescents’ recovery from SUDs, further research is warranted to better understand parental risk and protective factors, as well as their interaction with other relevant social-ecological risk factors (e.g., peer and community factors). Identifying the contexts in which opioid misuse is likely to arise among adolescents with SUDs can inform targeted prevention efforts for this population.

### Study Aims and Hypotheses

The current study examined risk and protective factors for opioid use in a sample of adolescents with histories of SUDs. Guided by ecological systems theory and prior research, we first examined associations between individual (mental health and substance use), microsystemic (peer perceptions of use, parent alcohol or drug [AOD] abuse history, and parenting behaviors), and exosystemic (academic performance, attitudes toward school, and perceived availability) risk factors and adolescent opioid use. We explored each risk and protective factor by assessing its unique association with opioid use within the broader social-ecology (individual, microsystem, and exosystemic domains). Second, to gain a better understanding of the role of parenting behaviors, we examined whether parental monitoring moderates any of the associations between these risk and protective factors and adolescent opioid use.

In line with these study aims, we hypothesized that each individual, microsystemic, and exosystemic risk factor would predict lifetime adolescent opioid use among a clinical sample of adolescents with SUD histories. We also hypothesized that parental monitoring would significantly moderate the associations between ecological risk factors and opioid use, such that greater levels of parental monitoring would buffer the relations between ecological risk factors and opioid use.

## Materials and Methods

### Participants and Procedure

We analyzed existing data from a longitudinal study that used a quasi-experimental design to examine the effects of post-SUD treatment schooling attendance on student outcomes (Finch et al., 2018). Adolescents and their families were recruited upon adolescents’ SUD treatment or continuing care programs (baseline assessment); a total of 294 adolescents and 323 parents enrolled in the study at baseline. Although the larger parent study included longitudinal follow-up assessments, the current manuscript analyzes data collected during only the baseline assessment to isolate study findings apart from any intervention effects. Adolescent participants identified as predominantly non-Hispanic white (74.9%) with ages ranging from 13 to 19 (*M* = 16.3 years, *SD* = 1.09) and were approximately equal in distribution by sex (50.2% male). For more information on sample characteristics, see Finch et al. (2018) and Tanner-Smith et al. (2018). All procedures followed were in accordance with the ethical standards of the University of Minnesota Institutional Review Board and with the Helsinki Declaration of 1975, as revised in 2000.

### Measures

#### Primary Outcome

##### Opioid Use

The outcome of interest in this study was measured using a single self-reported dichotomous item about adolescents’ lifetime opioid misuse at baseline–“Have you ever used any of these drugs: Opioids/Narcotics (heroin, smack, morphine, codeine, Demerol, methadone, opium, Vicodin, Oxycontin, and oxycodone)?” This outcome item was coded as *yes* (1) or *no* (0).

#### Individual-Level Predictors

##### Mental Health

Several mental health constructs were assessed as individual-level risk factors for the current study. We used the M.I.N.I. Structured Clinical Interview (M.I.N.I.-SCID), a brief structured diagnostic interview for major psychiatric disorders derived from the symptomology defined by the DSM-IV and ICD-10, to examine adolescents’ self-reported mental health symptoms of major depressive disorder (MDD), generalized anxiety disorder (GAD), and post-traumatic stress disorder (PTSD), as well as antisocial traits (Sheehan et al., 1999). This measure assessed whether adolescents experienced any symptoms of each diagnosis in the 12 months prior to enrolling in the substance use treatment program (*yes/no*). Antisocial traits were assessed by whether adolescents met the point-in-time clinical threshold of DSM-IV symptoms of antisocial personality disorder (*yes/no*). These measures do not represent a formal clinical diagnosis; rather, they assessed whether adolescents self-reported any symptoms for MDD, GAD, and PTSD, and whether adolescents reported antisocial traits at or above a clinically indicated threshold (i.e., at least three antisocial traits based on DSM-IV criteria).

##### Substance Use

Tobacco, marijuana, and alcohol use were examined as individual-level risk factors. Tobacco use was assessed through a single binary item (*yes/no*) asking, “in the past 12 months, have you used tobacco products, including cigarettes, cigars, a pipe, or chewing tobacco/snuff?” Marijuana and alcohol use were also measured with two binary items (*yes/no*) indicating whether adolescents reported using marijuana in the past year or using alcohol to the point of intoxication in the past year, respectively.

#### Familial- and Peer-Level Predictors

##### Parenting Practices

Parenting practices were measured using a shortened version (15 items) of the original 42-item parent-reported Alabama Parenting Questionnaire (PAPQ) (Frick, 1991). The PAPQ includes measures of three subscales of parenting practices: positive parenting (six items), poor parental monitoring (five items), and inconsistent discipline (four items). Response options used a five-point Likert scale ranging from *Never* (1) to *Always* (5), where parents rated the frequency of parenting in the past 12 months. An example item for poor parental monitoring was, “Your child fails to leave a note or let you know where he/she is going.” Scores for the three subscales were determined by calculating the mean for each subscale. Higher mean scores on each subscale indicate higher levels of each parenting construct. The PAPQ subscales have shown strong concurrent and predictive validity in a prior study with this sample (Nichols et al., Under review). The current sample showed adequate internal consistency in the three subscales: positive parenting (α = 0.78), inconsistent discipline (α = 0.70), and poor parental monitoring (α = 0.74).

##### Parent With Alcohol or Drug Abuse History

One dichotomous (*yes/no*) adolescent-reported item was used to measure parents’ alcohol or drug abuse history: “Do either of your biological parents have a history of an AOD abuse problem?”

##### Peer Attitudes Scale

Substance approving peer attitudes were assessed using 13 items from the Personal Experiences Inventory (Winters and Henley, 1989). Response options were measured on a four-point Likert scale, with responses ranging from *Strongly disagree* (1) to *Strongly agree* (4), where responses were anchored to the time in the adolescent’s life when they were using drugs at their heaviest level. An example item was, “My friends think that using drugs or alcohol makes hanging out more fun.” A mean score for peer attitudes was determined by calculating the mean of the 13 items, with higher scores indicating higher peer approval of substance use. This measure demonstrated good internal consistency in the analytic sample (α = 0.87).

#### School-/Community-Level Predictors

##### Academic Performance

Grade point average (GPA) was used to assess adolescents’ academic performance. One continuous adolescent-reported item measured adolescents’ most recent GPA, on a scale ranging from 0 to 4.

##### Perceived Availability of Substances

Perceived availability of alcohol, marijuana, prescription drugs, other illicit drugs, and over-the-counter drugs was measured using a modified version of Monitoring the Future’s Perceived Availability of Drugs Scale (Bachman et al., 2001). Survey questions began with one question “How difficult do you think it would be for you to get each of the following drugs, if you wanted some?” and listed multiple substance types. Response options were measured on a five-point Likert scale ranging from *Probably impossible* (1) to *Very easy* (5). A mean score was computed for each participant, where higher values represent greater overall perceived availability of drugs and alcohol. This measure demonstrated adequate internal consistency in the current sample (α = 0.67).

##### Attitudes Toward School

Adolescents’ attitudes toward school were measured using 10 items from the Behavior Assessment System for Children (BASC) (Reynolds and Kamphaus, 1992). Response options were *True/False* with the following prompt: “Thinking back to before you were in treatment, when you were using drugs the heaviest, click on the “True” option if you agree with the sentence or click on “False” if you don’t agree.” An example item was, “I can hardly wait to quit school.” The 10 items were added together to create a sum score, with higher scores representing higher negative attitudes toward school. The BASC demonstrated adequate internal consistency among the current sample (α = 0.75).

### Analytic Plan

To address the current study’s aims, we estimated a series of logistic regression models to examine the magnitude of associations between the individual, interpersonal, and school/community risk and protective factors and the odds of adolescent opioid use. All models adjusted for adolescent’s sex, race/ethnicity, whether they lived in a two-parent household, and whether they were enrolled in an RHS vs. a more traditional, non-RHS. First, a hierarchical logistic regression was conducted to examine the association between risk and protective factors of all the domains and adolescent opioid use. The first step of the hierarchical model examined associations between covariates and lifetime opioid use. The following step included all individual-level variables as predictors of adolescent opioid use. The third step in the model examined peer and parental risk and protective factors on opioid use while adjusting for individual-level predictors and covariates. The final step of the model examined the associations between school-/community-level predictors and adolescent opioid use, while adjusting for covariates and individual-, peer-, and parental-level risk and protective factors.

To address the second study aim, we added a multiplicative interaction term to test whether poor parental monitoring moderated the effect of each risk and protective factor on the odds of adolescent opioid use. When an interaction was tested (e.g., MDD symptoms and poor parental monitoring), all risk and protective factors were included in the model, as well as covariates. Results are presented as logit coefficients (*b*) from the logistic regression models, alongside corresponding odds ratio (*OR*) or adjusted odds ratio (*AOR*) effect sizes and their 95% confidence intervals. Model fit for each logistic regression tested was assessed using the Akaike Information Criterion (AIC).

There was a modest amount of missing data due to participant non-response and study attrition; missingness ranged from 5 to 24% among the variables of interest. Missing data were addressed using multiple imputation by chained equations (van Buuren and Groothuis-Oudshoon, 2011) to create 30 multiply imputed datasets with 30 iterations. All reported model estimates were obtained by pooling results across the imputed datasets using Rubin’s (1987) rules. All analyses were conducted using R 4.0.3 (R Core Team, 2020).

## Results

Table 1 shows the descriptive statistics for study variables included in the analyses. About 50% identified as male and approximately 75% identified as white. Less than one-half of the sample (47.4%) stated that they were enrolled in an RHS and approximately 36% of the adolescents in the sample stated they lived in a two-parent household. Approximately 67% of adolescents reported lifetime opioid use. Regarding mental health symptoms in the past 12 months, 31.6% of adolescents reported experiencing symptoms of MDD, 28.5 and 10.8% of adolescents reported experiencing symptoms of GAD and PTSD, respectively, and 39% of adolescents endorsed antisocial traits. Most adolescents reported at least some use of tobacco (84.2%), alcohol (69%), and marijuana (77.4%) in the past year. Approximately 57% of the sample reported a parent with past AOD abuse.

**TABLE 1:** Descriptive statistics for covariates, individual-, peer-, parental-, school-/community-level domains, and opioid use (*N* = 294).

Variable	*M* (*SD*)	Range	*n* (%)
Ever used opioids (1 = yes)			216 (66.9%)
Male (1 = yes)			162 (50.2%)
White (1 = yes)			242 (74.9%)
RHS enrollment (1 = yes)			153 (47.4%)
Two-parent household (1 = yes)			116 (35.9%)
MDD symptoms (1 = yes)			102 (31.6%)
GAD symptoms (1 = yes)			92 (28.5%)
PTSD symptoms (1 = yes)			35 (10.8%)
Antisocial traits (1 = yes)			126 (39%)
PY Tobacco use (1 = yes)			272 (84.2%)
PY Alcohol use (1 = yes)			223 (69%)
PY Marijuana use (1 = yes)			250 (77.4%)
Positive parenting	3.96 (0.59)	(1–5)	
Inconsistent discipline	2.70 (0.76)	(1–5)	
Poor parental monitoring	2.58 (0.81)	(1–5)	
Parent with past AOD abuse (1 = yes)			183 (56.7%)
Peer attitudes	3.05 (0.52)	(1–4)	
GPA	2.56 (0.87)	(0–4)	
Negative attitudes toward school	5.69 (2.60)	(0–10)	
Perceived availability	4.33 (0.59)	(1–5)	

*RHS = Recovery High School; MDD = Major Depressive Disorder; GAD = Generalized Anxiety Disorder; PTSD = Post-traumatic Stress Disorder; PY = Past year; AOD = alcohol or drug; GPA = Grade point average; M = mean; SD = Standard Deviation; n = number of observations.*

*Standard deviations are in parentheses. Percentages of adolescents that stated yes for each variable is reported in parentheses.*

Table 2 presents the findings from the hierarchical logistic regression models. In the covariate model, there was no evidence of significant associations between being male, white, enrolled in RHS, and living in a two-parent household with engaging in opioid use. The inclusion of individual-level risk factors in the subsequent model indicated that adolescents who endorsed antisocial traits had three times the odds of engaging in opioid use than adolescents who did not (AOR = 3.01, *p* < 0.001, 95% CI [1.55, 5.86])^1^. Experiencing MDD symptoms, GAD symptoms, or PTSD symptoms in the last 12 months were not significantly associated with engagement in opioid use. Use of tobacco, alcohol, and marijuana in the past year were also not significantly associated with engagement in opioid use. After adding parent and peer risk and protective factors, the model showed that having a parent with past AOD abuse was associated with an 87% increase in the odds of engaging in opioid use (AOR = 1.87, *p* = 0.04, 95% CI [1.04, 3.39]) when adjusted for other individual, parent, and peer predictors. Other parental dimensions, including poor parental monitoring, inconsistent discipline, and positive parenting, were not significantly associated with engagement in opioid use. Similarly, peer attitudes did not show evidence of a significant association with engagement in opioid use. In the final model, including school-level and community-level predictors, the community-level predictor (perceived availability of substances) was significantly associated with ever using opioids (AOR = 1.90, *p* = 0.02, 95% CI [1.12, 3.20]). School-level predictors, including GPA and negative attitudes toward school, however, were not significantly associated with engagement in opioid use. A significant association was found for adolescents with a two-parent household having higher odds of engaging in engaging in opioid use (AOR = 2.09, *p* = 0.038, 95% CI [1.04, 4.20]) when including the school- and community-level predictors. As seen in Table 2, both parent with a past AOD abuse and antisocial traits remained significantly associated with engaging in opioid use when including additional ecological predictors in subsequent models.

**TABLE 2:** Hierarchical logistic regression of individual-, parent- and peer, school-/community-level predictors of opioid use.

Variable	Covariates		Individual		Parent/Peer		School/Community	
	*b (SE)*	OR [95% CI]	*b (SE)*	AOR [95% CI]	*b (SE)*	AOR [95% CI]	*b (SE)*	AOR [95% CI]
Male	0.15 (0.30)	1.16 [0.64, 2.11]	0.21 (0.30)	1.23 [0.68, 2.23]	0.18 (0.31)	1.20 [0.65, 2.20]	0.11 (0.32)	1.12 [0.60, 2.09]
White	0.55 (0.41)	1.74 [0.76, 3.96]	0.68 (0.40)	1.97 [0.90, 4.31]	0.60 (0.40)	1.82 [0.82, 4.03]	0.64 (0.41)	1.89 [0.84, 4.27]
RHS enrollment	0.47 (0.28)	1.59 [0.81, 2.78]	0.39 (0.32)	1.48 [0.79, 2.77]	0.45 (0.32)	1.57 [0.83, 2.96]	0.33 (0.33)	1.38 [0.73, 2.63]
Two-parent household	0.52 (0.30)	1.69 [0.93, 3.05]	0.48 (0.32)	1.62 [0.87, 3.03	0.62 (0.34)	1.86 [0.95, 3.63]	0.74 (0.35)*	2.09 [1.04, 4.20]
MDD symptoms			0.00 (0.35)	1.00 [0.50, 2.00]	0.02 (0.36)	1.02 [0.50, 2.07]	0.03 (0.37)	1.03 [0.50, 2.13]
GAD symptoms			0.48 (0.338)	1.62 [0.77, 3.41]	0.52 (0.39)	1.68 [0.78, 3.61]	0.51 (0.39)	1.67 [0.77, 3.63]
PTSD symptoms			0.27 (0.53)	1.31 [0.46, 3.71]	0.22 (0.53)	1.24 [0.43, 3.56]	0.09 (0.54)	1.10 [0.38, 3.18]
Antisocial traits			1.10 (0.34)**	3.01 [1.55, 5.86]	1.12 (0.35)**	3.05 [1.54, 6.04]	0.98 (0.35)*	2.65 [1.32, 5.32]
PY Tobacco use			0.47 (1.10)	1.61 [0.17, 15.20]	0.42 (0.35)	1.52 [0.14, 16.20]	0.27 (1.19)	1.31 [0.12, 14.9]
PY Alcohol use			0.27 (0.42)	1.31 [0.58, 2.99]	0.28 (0.42)	1.32 [0.57, 3.04]	0.15 (0.44)	1.17 [0.49, 2.76]
PY Marijuana use			0.08 (0.50)	1.08 [0.40, 2.91]	0.18 (0.50)	1.19 [0.44, 3.22]	0.24 (0.52)	1.27 [0.45, 3.54
Positive parenting					0.09 (0.26)	1.09 [0.66, 1.81]	0.10 (0.26)	1.11 [0.66, 1.85]
Inconsistent discipline					0.10 (0.21)	1.11 [0.74, 1.66]	0.08 (0.21)	1.08 [0.72, 1.64]
Poor parental monitoring					−0.05 (0.20)	0.96 [0.65, 1.40]	0.03 (0.20)	1.03 [0.69, 1.54]
Parent with past AOD abuse					0.63 (0.30)*	1.87 [1.04, 3.39]	0.67 (0.31)*	1.95 [1.05, 3.59]
Peer attitudes					−0.09 (0.29)	0.91 [0.51, 1.63]	−0.30 (0.32)	0.74 [0.39, 1.40]
GPA							0.12 (0.20)	1.13 [0.76, 1.69]
Negative attitudes toward school							0.06 (0.06)	1.06 [0.94, 1.21]
Perceived availability							0.64 (0.27)*	1.90 [1.12, 3.20]
Likelihood ratio test statistic			χ2 = 1.45		χ2 = 0.92		χ2 = 2.10	
AIC	376.24		366.91		370.59		366.76	

*RHS = Recovery High School; MDD = Major Depressive Disorder; GAD = Generalized Anxiety Disorder; PTSD = Post-traumatic Stress Disorder; PY = Past year; AOD = alcohol or drug; GPA = Grade point average; b = Unstandardized logit coefficient; SE = Standard errors; AOR = Adjusted odds ratio; OR = Odds ratio; CI = Confidence interval; AIC = Akaike Information Criterion.*

Standard errors are in parentheses. All models adjusted for covariates.

*p < 0.05; **p < 0.01.

### Potential Moderating Effect of Poor Parental Monitoring With Individual-Level Predictors

As shown in Table 3, there was no evidence that poor parental monitoring significantly moderated the association between the individual-level predictors and opioid use. Indeed, the interaction between MDD symptoms and poor parental monitoring was not significantly associated with odds of adolescents ever using opioids (AOR = 1.25, *p* = 0.60, 95% CI [0.54, 2.90]). Similarly, there was no evidence that poor parental monitoring moderated the associations between other mental health constructs, including GAD symptoms (AOR = 2.24, *p* = 0.12, 95% CI [0.81, 6.20]), PTSD symptoms (AOR = 1.23, *p* = 0.74, 95% CI [0.35, 4.27]), and antisocial traits (AOR = 1.16, *p* = 0.69, 95% CI [0.55, 2.45]), with using opioids. Finally, there was no evidence that poor parental monitoring moderated the associations between other individual-level predictors and adolescents’ opioid use: tobacco use (AOR = 0.82, *p* = 0.90, 95% CI [0.04, 15.60]), alcohol use (AOR = 1.17, *p* = 0.75, 95% CI [0.45, 3.02]), marijuana use (AOR = 1.92, *p* = 0.18, 95% CI [0.73, 5.05]).

**TABLE 3:** Moderation analyses of individual-level predictors and poor parental monitoring on opioid use.

	**MDD symptoms**		**GAD symptoms**		**PTSD symptoms**		**Antisocial traits**	
**Effect**	**b (SE)**	**AOR [95% CI]**	**b (SE)**	**AOR [95% CI]**	**b (SE)**	**AOR [95% CI]**	**b (SE)**	**AOR [95% CI]**

Main effect	0.04 (0.37)	1.04 [0.50, 2.16]	0.60 (0.42)	1.82 [0.80, 4.14]	0.11 (0.54)	1.12 [0.38, 3.27]	0.98 (0.35)*	2.66 [1.32, 5.33]
Poor parental monitoring	−0.05 (0.24)	0.95 [0.59, 1.53]	−0.13 (0.22)	0.88 [0.56, 1.36]	0.01 (0.21)	1.01 [0.67, 1.53]	−0.03 (0.26)	0.97 [0.58, 1.61]
Interaction	0.22 (0.43)	1.25 [0.54, 2.90]	0.81 (0.51)	2.24 [0.81, 6.20]	0.21 (0.63)	1.23 [0.35, 4.27]	0.15 (0.38)	1.16 [0.55, 2.45]
AIC	368.13		365.00		368.41		368.47	

	**Past Year Tobacco Use**		**Past Year Alcohol Use**		**Past Year Marijuana Use**			
**Effect**	**b (SE)**	**AOR [95% CI]**	**b (SE)**	**AOR [95% CI]**	**b (SE)**	**AOR [95% CI]**		

Main effect	0.09 (1.97)	1.09 [0.02, 58.90]	0.16 (0.44)	1.18 [0.49, 2.81]	0.34 (0.54)	1.41 [0.49, 4.06]		
Poor parental monitoring	0.10 (0.26)	1.11 [0.66, 1.85]	0.10 (0.26)	1.11 [0.66, 1.86]	0.12 (0.26)	1.13 [0.68, 1.89]		
Interaction	−0.20 (1.47)	0.82 [0.04, 15.60]	0.15 (0.48)	1.17 [0.45, 3.02]	0.65 (0.49)	1.92 [0.73, 5.05]		
AIC	367.86		368.22		366.37			

MDD = Major Depressive Disorder; GAD = Generalized Anxiety Disorder; PTSD = Post-traumatic Stress Disorder; b = Unstandardized logit coefficient; SE = Standard errors; AOR = Adjusted odds ratio; CI = Confidence interval; AIC = Akaike Information Criterion. Standard errors are in parentheses. All models adjusted for covariates.

*p < 0.05.

### Potential Moderating Effect of Poor Parental Monitoring With Parental-/Peer-Level Predictors

Table 4 shows that there was no evidence that poor parental monitoring significantly moderated the association between parental- and peer-level predictors and adolescent opioid use. Specifically, there was no evidence of a significant association between the interaction of positive parenting and poor parental monitoring with adolescents ever using opioids (AOR = 1.09, *p* = 0.77, 95% CI [0.61, 1.94]). Similarly, there was no evidence that poor parental monitoring moderated the associations between the other parental constructs, including inconsistent discipline (AOR = 1.18, *p* = 0.46, 95% CI [0.76, 1.81]) and having parents with histories of AOD abuse (AOR = 0.94, *p* = 0.87, 95% CI [0.45, 1.97]) with opioid use. Lastly, the interaction between peer attitudes and poor parental monitoring was not significantly associated with odds of adolescents ever using opioids (AOR = 1.30, *p* = 0.48, 95% CI [0.62, 2.76]).

**TABLE 4:** Moderation analyses of parental- and peer-level predictors and poor parental monitoring on opioid use.

	Positive Parenting		Inconsistent Discipline		Parent with Past AOD Abuse		Peer Attitudes	
Effect	*b (SE)*	AOR [95% CI]	*b (SE)*	AOR [95% CI]	*b (SE)*	AOR [95% CI]	*b (SE)*	AOR [95% CI]
Main effect	0.10 (0.26)	1.11 [0.66, 1.86]	0.05 (0.21)	1.06 [0.69, 1.61]	0.66 (0.31)*	1.94 [1.05, 3.59]	−0.29 (0.32)	0.75 [0.40, 1.42]
Poor parental monitoring	0.03 (0.20)	1.03 [0.69, 1.54]	0.03 (0.20)	1.03 [0.69, 1.54]	0.07 (0.31)	1.07 [0.58, 1.98]	0.04 (0.21)	1.05 [0.70, 1.57]
Interaction	0.09 (0.29)	1.09 [0.61, 1.94]	0.16 (0.22)	1.18 [0.76, 1.81]	−0.06 (0.38)	0.94 [.045, 1.97]	0.27 (0.38)	1.30 [0.62, 2.76]
AIC	368.47		368.07		368.58		368.04	

b = Unstandardized logit coefficient; SE = Standard errors; AOD = alcohol or drug; OR = Odds ratio; CI = Confidence interval; AIC = Akaike Information Criterion. Standard errors are in parentheses. All models adjusted for covariates.

*p < 0.05.

### Potential Moderating Effect of Poor Parental Monitoring With School-/Community-Level Predictors

Table 5 shows the interaction findings between school-/community-level predictors and poor parental monitoring with adolescents ever using opioids. There was no evidence that poor parental monitoring significantly moderated the associations between GPA, negative attitudes toward school, and perceived availability with ever engaging in opioids. Specifically, there was no evidence that poor parental monitoring significantly moderated the association between GPA and adolescents ever using opioids (AOR = 0.95, *p* = 0.86, 95% CI [0.56, 1.62]), nor between negative attitudes toward school and opioid use (AOR = 1.14, *p* = 0.07, 95% CI [0.99, 1.32]). Finally, there was no evidence of a significant association between the interaction of perceived availability and poor parental monitoring with adolescents ever using opioids (AOR = 1.53, *p* = 0.18, 95% CI [0.82, 2.85]).

**TABLE 5:** Moderation analyses of school-/community-level predictors and poor parental monitoring on opioid use.

	GPA		Perceived Availability		Negative Attitudes Toward School	
Effect	*b (SE)*	AOR [95% CI]	*b (SE)*	AOR [95% CI]	*b (SE)*	AOR [95% CI]
Main effect	0.13 (0.20)	1.14 [0.76, 1.70]	0.61 (0.27)*	1.84 [1.08, 3.13]	0.07 (0.07)	1.07 [0.94, 1.22]
Poor parental monitoring	0.03 (0.20)	1.03 [0.69, 1.54]	0.06 (0.21)	1.07 [0.70, 1.61]	0.04 (0.21)	1.04 [0.68, 1.58]
Interaction	−0.05 (0.27)	0.95 [0.56, 1.62]	0.42 (0.32)	1.53 [0.82, 2.85]	0.13 (0.07)	1.14 [0.99, 1.32]
AIC	368.17		366.33		364.65	

b = Unstandardized logit coefficient; SE = Standard errors; AOR = Adjusted odds ratio; CI = Confidence interval; AIC = Akaike Information Criterion. Standard errors are in parentheses. All models adjusted for covariates.

*p < 0.05.

## Discussion

This study examined several social-ecological risk and protective factors associated with lifetime opioid use among a sample of adolescents with histories of SUDs. Our results suggest that opioids are a commonly used illicit substance among this clinical adolescent sample, evidenced by the 67% of adolescents reporting lifetime opioid use. This prevalence rate is comparable to previous findings of opioid use rates among adolescents in recovery from SUDs (Vosburg et al., 2016; Tanner-Smith et al., 2018), highlighting the generalizability of opioid use characteristics among high-risk clinical populations of adolescents. Our hypothesis that risk factors at each social-ecological level would significantly predict lifetime opioid use was partially supported. Regarding the role of family and parenting contexts, our results demonstrated that adolescents whose parents have a history of AOD abuse were more likely to report ever using opioids compared to those who did not report a parental substance use history. As hypothesized, adolescents who endorsed antisocial traits also had greater odds of reporting lifetime opioid use compared to adolescents who did not meet this threshold. This finding is consistent with prior research linking antisocial behavior to adolescent opioid misuse (Sung et al., 2005; Nargiso et al., 2015; Vaughn et al., 2016; Griesler et al., 2019). Additionally, adolescents who reported greater perceived availability of substances had greater odds of reporting lifetime opioid use compared to adolescents with lower perceived availability of substances. We found no evidence that adolescents’ past year substance use (tobacco, marijuana, or alcohol) was associated with their lifetime opioid use, nor any evidence that adolescents’ prior mental health symptoms of MDD, GAD, or PTSD, nor peer attitudes favorable toward drugs, were predictive of lifetime opioid use. Given that previous studies have consistently reported significant associations between substance use and mental health histories and subsequent opioid use outcomes (Barnett et al., 2019; Griesler et al., 2019; Bhatia et al., 2020; Bonar et al., 2020), further research is warranted to replicate the null findings reported herein.

These results highlight the potentially impactful role of parental substance use histories on adolescent opioid use. The family context is incredibly influential during the developmental stage of adolescence, underlying the significance of understanding the development and progression of SUDs among adolescents, particularly among those with parents who have existing substance use-related concerns and histories (Chassin and Handley, 2006). Prior research has documented that parental SUDs increase the likelihood that their children will develop SUDs (Biederman et al., 2000). Moreover, effects of protective parenting behaviors on children’s outcomes might be diminished among parents with SUDs compared to parents without substance use problems (Arria et al., 2012). Family and parenting characteristics therefore affect adolescents’ behaviors both directly and indirectly, highlighting the complex nature of parenting when substance use is a factor within the family context. Growing behavioral genetics research suggests that substance use during adolescence is heavily influenced by environmentally mediated factors, including parent–child relationship problems and peer deviance, which influence adolescent phenotypes, over and beyond heritable biological influences alone (Walden et al., 2004). Although parental substance abuse was examined as a microsystemic predictor of opioid use, future research should consider examining this variable as a possible proxy of biological vulnerability for addiction or substance use among adolescents. Such an investigation may provide more nuance to the complex nature of substance use in the context of family and parents.

The hypothesis that level of parental monitoring would moderate associations between social-ecological risk factors and opioid use was not supported in the current study. We found no evidence that parental monitoring levels significantly moderated associations between social-ecological risk factors and adolescents’ lifetime opioid use. These null results could be due to limited statistical power using our analytic sample of 294 adolescents. Future research should thus attempt to replicate this effect in larger samples of adolescents with SUD histories and similar risk profiles as the current sample. These null findings might also reflect a lack of nuance and sensitivity in our measure of parental monitoring (see Kerr and Stattin, 2000; Stattin and Kerr, 2000; Kerr et al., 2010), despite its demonstrated predictive validity among other samples of adolescents (Elgar et al., 2007; Zlomke et al., 2014, 2015; Gross et al., 2017). Historically, parental monitoring has been conceptualized as an active attempt by parents to monitor and follow the whereabouts of their children. However, this parental management strategy has been found to be most effective in the context of positive parent-adolescent relationships that would evoke adolescent self-disclosure of information and risk behaviors (Stattin and Kerr, 2000; Fletcher et al., 2004; Keijsers et al., 2009; Rusby et al., 2018). Indeed, adolescent self-disclosure is an important component of parental monitoring (Kerr and Stattin, 2000; Stattin and Kerr, 2000; Rusby et al., 2018), supporting the need to understand the relationship quality alongside factors such as conflict and communication. Thus, family focused interventions with adolescents with SUD histories may need to consider the way in which parental monitoring is being assessed. This may be an important area for prevention among adolescents with histories of SUDs.

Our results demonstrate the applicability of studying adolescents’ perceived availability of substances (at the exosystem level), parent’s substance use (microsystem level), and antisocial traits (individual level) among students in recovery from SUDs. Some theoretical frameworks, such as the recovery capital framework (Granfield and Cloud, 1999; Hennessy, 2017), highlight how access to and accumulation of resources across multiple ecological levels can aid the substance use recovery process. Continuing care options that address the multiple social-ecological needs of youth in recovery, are therefore likely to successfully support youths’ recovery needs. For example, RHSs, which aim to support students’ social and community capital by fostering social connectedness with sober peers, supportive school staff, and family members, have shown positive effects in prolonging abstinence from substance use during recovery (Finch and Karakos, 2014; Finch et al., 2018; Tanner-Smith et al., 2019, 2020). Other approaches drawing on integrated and holistic care models providing tailored therapeutic services to adolescents in recovery from SUDs (e.g., Latimer et al., 2000) may thus be similarly effective in addressing the numerous issues facing these adolescents.

### Limitations

The findings from the current study should be considered alongside several study limitations. First, because we relied on existing data, we were only able to study the outcome of interest–opioid use–using one binary item. This item inherently limited our ability to examine predictors of the frequency or severity of adolescent opioid use. Future research studies in samples of adolescents with SUDs should collect more nuanced data about opioid misuse to better understand predictors of both the likelihood and extent of opioid use (e.g., Boyd et al., 2006). There were additional limitations due to measures used in the current study that are important to note. It is possible that there was insensitive measurement bias if the measures were not developmentally appropriate for this sample of adolescents. Additionally, it is possible that opioid use was under-reported in the present sample, as well as other national samples of adolescents (Palamar et al., 2016a); a possible source of attention bias. Given that adolescents had recently been discharged from SUD treatment, it is possible that some participants felt pressure to respond favorably to the questionnaire items regarding drug use. Second, given the small and relatively homogenous sample (in terms of race/ethnicity and socioeconomic status), future research should aim to study these ecological risk and protective factors in larger clinical samples of adolescents from more diverse backgrounds. Finally, given our reliance on previously collected data, there were several potential confounding variables highlighted in the literature that were not included in our final analytic models, such as adolescents’ sensation-seeking and self-medication motives (Khantzian, 1997; Boyd et al., 2006, 2009; Young A. et al., 2012; Romer et al., 2017). Similarly, the scope of this study did not include examining potential mediators; however, prior research suggests these associations may hold additional complexity that should be further explored. For instance, prior studies have demonstrated that positive parental involvement may act as a mediator between parent characteristics such as SUD history on youth psychosocial outcomes, which may include adolescent opioid and other substance use (Bijttebier et al., 2006; Burstein et al., 2006). Future research is thus warranted to examine possible differences in motivations for opioid use among adolescents with SUD histories as well as potential mediators that may elucidate the mechanisms underlying the link between various risk factors and adolescent opioid misuse.

## Conclusion

This study adds to the empirical evidence base on adolescent opioid misuse in several important ways. First, this is the first study to our knowledge that uses a social-ecological framework to study risk and protective factors of opioid use among adolescents with a history of SUDs. Examining these associations in this understudied clinical population is critical for promoting positive outcomes among adolescents after they are discharged from formal substance use treatment. High school students with histories of SUDs represent a high-risk clinical subpopulation for problematic substance use and relapse. More research is needed on the social epidemiology of substance use–and opioid use, more specifically–in this population, which can be used to inform efficacious and targeted preventive and continuing care interventions for these adolescents. Continuing care programs that offer individualized treatment plans should concentrate on the important roles that families, peers, and school environment have in promoting positive outcomes among adolescents with histories of SUDs and opioid misuse.

## Data Availability Statement

The data analyzed in this study is subject to the following licenses/restrictions: Sensitive data that cannot be publicly shared. Requests to access these datasets should be directed to ET-S, etanners@uoregon.edu.

## Ethics Statement

The studies involving human participants were reviewed and approved by University of Minnesota Institutional Review Board. Written informed consent to participate in this study was provided by the participants’ legal guardian/next of kin.

## Author Contributions

LN: conceptualization, writing—original draft, writing—review and editing, and project administration. JP: conceptualization, methodology, formal analysis, and writing—review and editing. CF: conceptualization, methodology, and writing—review and editing. KO’B: conceptualization and writing—review and editing. ET-S: conceptualization, investigation, resources, writing—review and editing, and supervision. All authors contributed to the article and approved the submitted version.

## Conflict of Interest

The authors declare that the research was conducted in the absence of any commercial or financial relationships that could be construed as a potential conflict of interest.

## Footnotes

1 *Post hoc* analyses excluding non-significant covariates were conducted to increase statistical power. Results of these *post hoc* analyses yielded no substantial or meaningful changes in model fit, statistical significance, or conclusions.
